# School-Level Factors within Comprehensive School Health Associated with the Trajectory of Moderate-to-Vigorous Physical Activity over Time: A Longitudinal, Multilevel Analysis in a Large Sample of Grade 9 and 10 Students in Canada

**DOI:** 10.3390/ijerph182312761

**Published:** 2021-12-03

**Authors:** Melissa Pirrie, Valerie Carson, Joel A. Dubin, Scott T. Leatherdale

**Affiliations:** 1 School of Public Health Sciences, University of Waterloo, Waterloo, ON N2L 3G1, Canada; jdubin@uwaterloo.ca (J.A.D.); sleatherdale@uwaterloo.ca (S.T.L.); 2 Faculty of Kinesiology, Sport, and Recreation, University of Alberta, Edmonton, AB T6G 2H9, Canada; vlcarson@ualberta.ca; 3 Department of Statistics and Actuarial Science, University of Waterloo, Waterloo, ON N2L 3G1, Canada

**Keywords:** gender, secondary school, framework, linear mixed effects model

## Abstract

(1) The majority of Canadian youth are insufficiently active, and moderate-to-vigorous physical activity (MVPA) decreases substantially during secondary school. School factors within the comprehensive school health (CSH) framework may help attenuate this decline. This study aimed to examine how youth MVPA changes over a three-year period and evaluate the school characteristics associated with preventing the decline in MVPA over time, guided by the CSH framework. (2) This study uses COMPASS survey data from 78 secondary schools in Ontario and Alberta that participated in Year 2 (2013/14), Year 3 (2014/15), and Year 4 (2015/16), and 17,661 students attending these schools. Multilevel (linear mixed effects) models were used to determine the association between school-level factors and student MVPA (weekly minutes) over time, stratified by gender. (3) Both male and female students had a significant decline in MVPA across the 3 years, with a greater decrease observed among female students. Within the CSH framework, the school’s social environment, partnerships, and policies were associated with student MVPA over time, however the specific school factors and directions of associations varied by gender. (4) School-based interventions (e.g., public health partnerships) may help avoid the decline in MVPA observed in this critical period and support student health.

## 1. Introduction

Secondary school is a critical life stage when physical activity (PA) declines sharply [1,2,3,4]. This substantial reduction in PA is concerning as there are numerous physical and mental health benefits for youth who are active, such as reduced risk of hypertension and diabetes [5,6]. In addition, PA during adolescence is significantly correlated with adult PA behaviour and health [7,8]. Because active youth are more likely to be active adults, this leads to many health benefits later in life as well, including reduced risk of some cancers, cardiovascular disease, stroke, and diabetes [6,7]. In particular, adult bone density is heavily dependent on PA during youth [7]. As a result, it is imperative to support youth in avoiding the ‘adolescent decline’ in PA during secondary school to realize both immediate and future health benefits.

The *Canadian 24-Hour Movement Guidelines for Children and Youth* [9] recommends that youth participate in at least 60 min of moderate-to-vigorous PA (MVPA) daily and describes that achieving additional MVPA is associated with even greater health benefits [9]. According to the combined cycle 2 (2009–2011) and cycle 3 (2012–2013) of the Canadian Community Health Measures Survey (CHMS), which objectively measured PA with accelerometers, only 31% of youth aged 12 to 17 years attained an average of 60 min of MVPA per day [10]. In addition, a significant decline in PA typically occurs around 15–16 years of age [3], and females are more likely to experience the PA decline earlier than males [4]. Adolescents spend a substantial amount of time at school each week and, although it is not their primary mandate, schools can play a critical role in supporting students in achieving optimal levels of MVPA during this pivotal period.

The comprehensive school health (CSH) framework [11] provides guidance on four inter-connected components on which schools can focus to improve health behaviours among their students: (1) social and physical environment; (2) partnerships and services; (3) teaching and learning; and (4) policy. The social and physical environment component represents the relationships between individuals (e.g., students, staff, family members), as well as the physical spaces and amenities available to support student health. Specific to PA, students are more likely to be active if they attend schools with higher ratings of social support [12,13], greater social cohesion [14], and more facilities for PA available [15,16,17]. A cluster-randomized controlled trial of a school-based PA intervention for female grade 9 students included changes to both the physical education curriculum and the social environment (e.g., teacher role modeling, PA promotion by the school nurse, and inclusion of family/community) [18]; however, there was no study arm that had the physical education curriculum changes only, and therefore it is not possible to discern if any of the observed increase in vigorous PA can be attributed to the social environment changes.

The partnerships and services component represents collaborations between the school and external organizations to support student health. There is very limited research on the potential impacts of these partnerships, however previous research in elementary schools has found that students in grades 5 to 8 attending schools with established community partnerships are more likely to be highly active [19]. 

The third component, teaching and learning, represents the school’s health-related curricular and extracurricular programming, as well as teaching training. This domain is where the majority of school-based interventions have focused their attention [20,21,22]. In general, curricular changes to provide additional minutes of physical education per week or change the content of the physical education curriculum have had positive impacts on student PA both within and outside of school hours [20,21,22,23,24,25,26]. In addition, offering extracurricular and intramural PA opportunities has been associated with higher PA [27,28], especially among female students [29,30]. 

Finally, the policy component represents the administrative policies and decision making that may impact student health behaviours. An earlier review article by Sallis et al. [3] in 2000 had found a positive association between access to facilities and student PA, but a subsequent review by Van Der Horst et al. [31] in 2007 found no association. A recent study of 34 schools found that students attending schools with a health policy and schools with a mobile phone ban during breaks had significantly higher minutes of MVPA during school hours [32].

Although many studies have examined one or a few school features found within the CSH framework that may improve PA behaviour, there was no study identified that examined school features within all four components of the CSH framework simultaneously and their association with the trajectory of MVPA in a cohort of students followed longitudinally. This is a substantial gap in the literature. There is a need to gain knowledge about the understudied components of CSH (e.g., partnerships and services), and also to better understand the school features within CSH that may be the most impactful by examining them concurrently in a single study.

The aims of the current study are to examine how youth MVPA changes over a three-year period in a large sample of secondary school students, stratified by gender, and to evaluate the school characteristics associated with preventing the decline in MVPA over time, guided by the CSH framework [33]. This novel study is exploratory and not seeking to confirm previous findings but instead will guide future CSH research specific to youth MVPA in a Canadian context.

## 2. Materials and Methods

### 2.1. Design

This study uses linked longitudinal data available from the COMPASS study [34]. COMPASS is a prospective cohort study (2012–2021) following a large sample of Canadian grade 9 to 12 students and the schools they attend, where data are to be used to evaluate natural experiments related to school-level impact on student health behaviours [34,35].

### 2.2. School and Student Participants

The current study examines data collected from 78 schools in Ontario (*n* = 69) and Alberta (*n* = 9) that provided data in Year 2 (Y_2_: 2013/2014), Year 3 (Y_3_: 2014/2015), and Year 4 (Y_4_: 2015/2016). This time period corresponds with the largest initial intake of schools to COMPASS (Y_1_ and Y_2_) and when these schools completed the baseline COMPASS School Policies and Practices Questionnaire. Although COMPASS includes all students (grades 9 to 12), this study only includes students in grades 9 or 10 during Y_2_ so they could be tracked over three years prior to graduating from grade 12 and leaving the cohort. Due to the anonymous longitudinal linkage procedure (see ‘Data Collection Procedures’ for more details), only students who participated in COMPASS for at least two of the three years (did not need to be consecutive years) were included in the Y_2_ to Y_4_ linked COMPASS cohort (*n* = 17,700). The current study sample was also restricted to students with complete data (sex, grade, ethnicity, and MVPA) for at least one of the study years (*n* = 17,661 students); of those included in the study, 45.4% (*n* = 8011) had complete data for all three years, 51.7% (*n* = 9138) for any two years, and 2.9% (*n* = 512) for only one year.

### 2.3. Recruitment

Detailed student and school recruitment methods have been published [34]. In summary, COMPASS approached school boards that allow active-information, passive-consent. With school board approval, individual schools were recruited and then students and parents were sent information with multiple mechanisms to opt-out. Students could decline to participate or withdraw at any time. All students attending these schools were invited to participate and less than 1% opted out annually. From these 78 schools, 79% of eligible students participated in Y_2_, 79% in Y_3_, and 80% in Y_4_. Ethics approval was received by the University of Waterloo Office of Research Ethics (Project #30118), University of Alberta Research Ethics Office (Project #00040729), and by each school board and/or school as required.

### 2.4. Data Collection Procedures

A COMPASS staff member was onsite to facilitate data collection and identify the most appropriate individual to complete the school-level survey. Students completed the student-level survey during a class period and placed it in a sealed envelope. An identification code was self-generated by the student based on a combination of facts specific to that student (e.g., second letter of first name). This allowed student data to be collected anonymously while also facilitating linkage across multiple COMPASS years. Successful linkage and inclusion in the longitudinal dataset required the student to be matched across at least two years of data, and the linkage rate is approximately 80% [36]. Further details on the approach to longitudinal data linkage are available [36,37].

### 2.5. Measures

#### 2.5.1. School Variables

School-level data were collected the first year that the school took part in COMPASS. The COMPASS School Policies and Practices Questionnaire collects information on the school’s facilities, policies, programs, and resources, and was completed by a school administrator or other person identified as most knowledgeable about these characteristics. The 2011 Canada Census and school postal code were matched to describe school location (e.g., urban) and neighbourhood socio-economic status (SES).

The social environment variables were school enrolment size (total number of students), neighbourhood SES (median household income), school priority of PA (ranked from first through tenth), and whether the school promoted PA events (yes/no). The physical environment variables were the school location (rural/small urban, medium urban, large urban) and the presence or absence of indoor facilities, outdoor facilities, a gymnasium, change rooms, curtains for changing, secure lockers in the change area, and showers. The partnerships and services variables were the presence or absence of a collaboration with organizations from six categories (public health, non-governmental organization. parks and recreation, youth organization, health and fitness club, consultant/specialist). The teaching and learning variables were whether the school offered any intramural PA opportunities and any non-competitive PA opportunities (none, intramural only, non-competitive only, or both). Finally, the policy variables included whether the school had a written PA policy, uses student data to plan, had policies that permitted student access to equipment and facilities during non-instructional time and after school hours, and the level of resources received from the school board to support student health (staff time, space, and annual budget).

#### 2.5.2. Student Variables

The COMPASS student questionnaire (Cq) was completed annually. The Cq was designed to collect health behaviour data from students within their school setting, taking into consideration the feasibility of administration within a class period and student confidentiality [34]. Moreover, the questions were purposefully selected to align with national guidelines and match other surveys, allowing for cross-study comparison.

Two questions from Cq were summed to determine weekly MVPA: (1) “Mark how many minutes of HARD physical activities you did on each of the last 7 days,” and (2) “Mark how many minutes of MODERATE physical activities you did on each of the last 7 days.” Students were first provided with the definition that hard PA includes “jogging, team sports, fast dancing, jump-rope, and any other PAs that increase your heart rate and make you breathe hard and sweat.” Subsequently, moderate PA was defined for students as, compared to hard PA, “lower intensity activities such as walking, biking to school, and recreational swimming.”

A validation study found that students tended to under-report their moderate PA and over-report their vigorous activity, however the validity improved when combined as MVPA (Pearson correlation, r = 0.31) and was similar to other self-report questionnaires [38]. Test-retest reliability for the combined MVPA measure was moderate (r = 0.68). In addition, student gender, grade, and ethnicity were collected using the Cq.

### 2.6. Statistical Analysis

Descriptive analysis was conducted for school and student characteristics. Three-level linear mixed effects models (3-level growth models) with the outcome of weekly MVPA minutes were run for each gender separately. First, null models were run to identify the school-level intraclass correlations (ICCs) for female students and male students. Second, time was added as a Level-1 variable to create the unconditional growth model. Third, student ethnicity and grade cohort were added at the student-level (i.e., Level 2) as fixed effects, based on the first reported value by the student. Finally, school-level variables were added as fixed effects at Level 3, including cross-level interactions for each school-variable with time. All analyses were conducted using SAS 9.04 and the MIXED procedure was used to analyze the linear mixed models. As this study was exploratory, the results section of this article is primarily focused on the factors within each CSH component that were found to be statistically significant, however all results are included in the tables and should be scrutinized.

## 3. Results

### 3.1. School-Level Characteristics

In the 78 schools, all or almost all had a gymnasium (98.7%), other indoor facilities (97.4%), change rooms (94.9%), outdoor facilities (98.7%), and intervarsity sports (100%), so these features were not included in any of the regression models. The frequencies for each of the remaining school characteristics included in the regression models are provided in Table 1.

### 3.2. Student-Level PA

Descriptive statistics for the student-level characteristics are provided in Table 2. The null 3-level linear mixed effects model for female students found that repeated measures within the same student accounted for 45.0% of variance and students within schools accounted for 1.9% of the variance in weekly MVPA. For male students, the null 3-level model found that repeated measures within the same student accounted for 46.0% of variance and students within schools accounted for 2.4% of the variance in weekly MVPA. This indicates that 1.9–2.4% of the variance in weekly MVPA minutes across the repeated measures from Y_2_ to Y_4_ was due to the school attended, above and beyond the individual student characteristics. When time was added to the model (see Table 2), it was found that weekly MVPA minutes significantly decreased across the three years for both female students (β = −39.89 min/week, 95%CI: [−46.48, −33.31]) and male students (β = −18.03, 95%CI: [−26.25, −9.81]).

### 3.3. Associations between School-Level Social and Physical Environment and Student PA

Fixed effects results from the final regression models are provided in Table 3. For both models, time was no longer a significant predictor (*p* > 0.05) after the cross-level interactions between school characteristics and time were added. Looking at the school’s social environment, there were no school-level factors associated with student baseline MVPA in Y_2_ but there were several factors significantly associated (*p* < 0.05) with changes in MVPA over time (i.e., the slope of MVPA over the study years). Female students had significant increases in MVPA across years if their school was moderately sized (501–1000 students), whereas male students had significant decreases in MVPA over time if they attended a larger school (≥1001 students). Female and male students showed a similar pattern of significant decreases in MVPA minutes across years as the SES of their school’s neighbourhood increased, with the exception of female students attending schools in the highest SES neighbourhoods, where weekly MVPA across years was similar to that observed in the lowest SES category. Female students had a significant decrease in MVPA over time if their school ranked PA as 4th–6th priority compared to 1st–3rd, whereas male students had significant increase in MVPA over time if their school ranked PA as 7th–10th priority compared to 1st–3rd. Finally, female students had significant decreases in MVPA over time if their school reported promoting PA events.

For the school physical environment, female students had significantly lower MVPA minutes at baseline if their school provided showers but there was no significant association between shower facilities and change in MVPA over time, indicating that this relationship remained constant over time. Similarly, male students had significantly lower MVPA minutes at baseline if their school was in a medium urban population centre and there was no change in this relationship over time.

### 3.4. Associations between School-Level Partnerships and Services and Student PA

Looking at partnerships and services, both female and male students attending schools partnered with a health and fitness club had significantly lower baseline MVPA levels (*p* < 0.05), however there was a significant annual increase in MVPA for male students attending schools partnered with these clubs. Additionally, a significant increase in MVPA over time was observed for female students attending schools partnered with a PA consultant and a decrease for schools partnered with public health units.

### 3.5. Associations between School-Level Teaching and Learning and Student PA

For the teaching and learning component of CSH, none of the school-level factors included within the current study were found to be significantly associated (*p* < 0.05) with the baseline MVPA of students, nor with a change in MVPA over time.

### 3.6. Associations between School-Level Policy and Student PA

Finally, there were many school-level factors within the policy component of CSH associated (*p* < 0.05) with student MVPA at baseline and over time. Although using data to plan was not associated with baseline MVPA, it was significantly associated with an increase in MVPA over time for male students.

Females attending schools that permitted access to outdoor facilities during non-instructional time had a significantly higher baseline MVPA but then a significant decrease in MVPA for each year. However, female students did have a significant increase in MVPA for each year if they attended a school that provided access to indoor facilities during non-instructional school time. Both female and male students had substantially higher baseline MVPA if their school sometimes or always provided access to equipment during non-instructional time. Whereas this relationship remained consistent over time for female students (i.e., no significant slope), male students had significant decreases in MVPA over time associated with their school providing equipment sometimes or always.

For female students, if their school received resources from the school board for student health, there was no significant association with baseline MVPA, however they did have significantly higher MVPA over time if their school received additional staff time or a budget over $1000 for health and significantly lower MVPA over time if their school received space for health. In contrast, male students had a significant increase in MVPA over time if their school received space. Finally, male students had significantly higher baseline MVPA if their school received a moderate budget for health ($1–$1000) but also a significant decrease in MVPA over time associated with a moderate budget.

## 4. Discussion

Although the school-level ICCs for this outcome were low (1.9–2.4%), school-based interventions that cause small shifts in student MVPA can have a large impact at the population level [39]. In this study we found that many school-level factors were associated with the trajectory of weekly MVPA minutes over time. As expected, MVPA declined significantly across the three years and MVPA in female students declined at a significantly faster rate than in male students [1,2,3,4]; for each additional year, females participated in MVPA for 40 fewer minutes per week, whereas male students participated in 18 fewer minutes per week. However, the findings of this study suggest that school-based interventions may help attenuate the speed of this annual decline or even prevent the decline.

For the first component of CSH, the social and physical environment, there were no associations observed between student MVPA and the physical school characteristics included in the current study (i.e., rurality, showers, and change rooms); however, many social school characteristics were significantly associated with the slope of student MVPA over time. For both female and male students, attending schools situated in wealthier neighbourhoods was related to greater declines in MVPA over time, even with adjustment for urban/rural location. A similar result was observed in a longitudinal study that objectively measured MVPA annually across four years for a sample of 533 students in grades 9 and 10 from 31 secondary schools in Manitoba, Canada [25]. As it was beyond the scope of the current study to examine the interactions between school-level factors, future research with COMPASS data into the differences in PA-related characteristics between schools in high versus low SES neighbourhoods are needed. In addition, investigation into the location of where the PA is taking place (i.e., at school or in other neighbourhood locations) may help to shed light on the observed attenuated decline in MVPA for students attending schools in low SES neighbourhoods.

Other social school characteristics associated with MVPA over time were the size of student body and the promotion of PA events. For male students, attending schools with a large enrolment (>1000 students) was associated with lower MVPA over time. Although it is expected that larger schools would have more PA offerings [40,41], there may be other social differences in large schools that impact participation, such greater competition for resources (e.g., limited spots on intramural teams) [41], more non-PA clubs in which to participate [41] (e.g., improv, robotics, debate, other special interests), or decreased social connectedness [42]. Low social cohesion has previously been associated with higher rates of physical inactivity in a large sample of secondary school students [14]. For female students, attending schools that promote PA events was associated with a greater decline in PA over time. Although seemingly counterintuitive, it is possible that the types of PA events being promoted or the method of promotion may not align with the needs of female students, and may even deter female students from participating.

For the second component of CSH, partnerships and services, there were different beneficial partnerships identified for male and female students. Female students had a substantial negative slope for MVPA over time if their school was partnered with a public health unit, which was not observed for male students. At the same time, females attending schools that were working with a PA consultant had significant positive slope in their MVPA. Together, these findings suggest that existing public health resources may not be adequately tailored for female students and that other PA consultants were better able to support schools in promoting PA among this at-risk group.

Male students had a significant positive slope for MVPA over time if their school was partnered with a health and fitness club. Interestingly, attending schools partnered with a health and fitness club was also associated with substantially lower MVPA at baseline for both male and female students. It may be that for schools where incoming grade 9 and 10 students frequently have very low PA, the schools have strategically partnered with a service that can offer access to PA equipment and programming. It may also be that schools with limited onsite facilities or equipment are more likely to seek partnerships with health and fitness clubs. Additional research using COMPASS data examining the interactions between the school characteristics (e.g., onsite facilities and external partnerships) is warranted to help understand the nature of this finding. Previous research in elementary schools had identified that having established partnerships can be beneficial for student PA [19], and the current study findings suggest that a similar benefit can be gained at the secondary school level; however, the type of organization is important for realizing these benefits.

For the teaching and learning component of CSH, a school’s offering of intramural or non-competitive PA options was not found to be associated with the slope of student MVPA over time for either male or female students. This was an unanticipated finding as providing additional opportunities for PA in schools has been found to increase MVPA among secondary school students [28], and, in particular, two previous research studies found that female students engage in more MVPA when there are non-competitive options available [29,30]. However, one of these studies was cross-sectional and examined the number of days per week with more than 60 min of MVPA [30], not total minutes of MVPA, and the other study was a focus group regarding the school factors that were perceived by students to impact their MVPA [29]. In addition, the current study included a substantial number of school characteristics in the models and these other school factors may have a greater impact on the MVPA of both male and female students over time.

For the policy component of CSH, there were many school factors associated with the slope of student MVPA. Male students attending schools that use data to plan had a significant positive slope in their MVPA over time, which suggests that schools have been successful in collecting the information needed for decision making and implementing changes based on that data. It is noteworthy that this finding was only for male students; further study is needed to determine whether schools need to collect additional information that is specific to the PA needs of female students, or if they are already collecting this information but are unable to enact the required changes. Looking at specific policies, providing access to indoor facilities during non-instructional time was found in this study to be associated with a positive slope in MVPA for female students. Unexpectedly, there was a negative MVPA slope for females attending schools that provided access to outdoor facilities during this time. Additionally, access to facilities after school hours was not associated with student MVPA at baseline or over time. These nuanced findings may explain the conflicting results between two previous review articles for the association between access to school facilities and student PA outcomes [3,31]. Although beyond the scope of this study, future research using COMPASS data could examine whether schools that provided access to outdoor facilities were less likely to provide access to other facilities (e.g., indoor spaces and equipment). In a Canadian context, winter weather can impact student PA [43] and having access to indoor resources during non-instructional time periods may be very important in supporting student PA.

Finally, staff and space resources provided by the school board for health purposes were associated with student MVPA over time. Receiving staff time for health and a budget for health (≥$1000) were both associated with a positive slope for MVPA over time among female students; however, it is unknown how the funding and staff time were allocated within the schools (e.g., staff time for professional development training versus staff implementing student programming). Similarly, additional space for health provided by the school board was associated with a positive slope in MVPA for male students and a negative slope for female students, but the use of this space is unknown. Further research into how the schools were using these resources could help determine the types of activities, equipment, additional staffing, or infrastructure improvements that were facilitated through this school board funding and whether allowing schools to choose how to utilize these funds provides flexibility in targeting their self-identified areas of need.

This study presents data from a very large sample of schools and students, multiple waves of data from the same students, and information collected from both the students and school simultaneously. The COMPASS prospective cohort provided the unique opportunity to evaluate the impact of many school characteristics, guided by the CSH framework, on student MVPA behaviour change over time. Although self-reported student PA data are a limitation, it is expected that any bias would be in the same direction and magnitude each year. As such, the absolute MVPA measures may be inflated, but observed relationships and the change over time would not be affected by this self-report bias. There were some unexpected results identified and using a mixed approach of quantitative and qualitative in future research could shed light on these relationships. Another limitation is that only the baseline school characteristics were included due to substantial missing data and inconsistencies in schools reporting changes in follow-up surveys. Future research is needed to evaluate the impacts of school-level changes over time on the trajectory of student MVPA.

## 5. Conclusions

In conclusion, it is well-established that student MVPA decreases substantially over time, but the current study findings indicate that secondary schools can have a role in attenuating this decline. Within the CSH framework, the school’s social environment, partnerships, and policies were all found to be associated with student MVPA over time, however the specific school factors and directions of associations varied between male and female students. As this study was exploratory, there are many opportunities for future researchers to better understand the underlying mechanisms of the observed relationships to inform future school programs, policies, and school board provided resources.

## Figures and Tables

**Table 1:** Baseline characteristics of schools participating in Year 2 (2013–2014), Year 3 (2014–2015), and Year 4 (2015–2016) of the COMPASS Study in Ontario and Alberta, Canada.

School Characteristics		*n* = 78 % (n)
**Social Environment**		
Enrolment	1–500	41.0 (32)
	501–1000	50.0 (39)
	≥1001	9.0 (7)
SES	25,000–50,000	9.0 (7)
	50,001–75,000	65.4 (51)
	75,001–100,000	21.8 (17)
	≥100,001	3.9 (3)
School priority of PA	1st–3rd	21.8 (17)
	4th–6th	44.9 (35)
	7th–10th	23.1 (18)
	Missing	10.3 (8)
Promote PA events	Yes	88.5 (69)
	No	10.3 (8)
	Missing	1.3 (1)
**Physical Environment**		
Location	Rural	1.3 (1)
	Small urban	43.6 (34)
	Medium urban	16.7 (13)
	Large urban	38.5 (30)
Curtains for changing	Both girls and boys	26.9 (21)
	Girls only	19.2 (15)
	Boys only	1.3 (1)
	None	43.6 (34)
	Missing	9.0 (7)
Secure lockers in change room	Yes	79.5 (62)
	No	19.2 (15)
	Missing	1.3 (1)
Showers available	Both girls and boys	88.5 (69)
	Girls only	0.0 (0)
	Boys only	2.6 (2)
	None	5.1 (4)
	Missing	3.9 (3)
**Partnerships and Services**		
Organizations providing support (check all that apply)	Local public health	52.6 (37)
	Non-governmental organization	60.3 (47)
	Parks and recreation	27.0 (21)
	Youth organizations	29.5 (23)
	Health and fitness club	50.0 (39)
	Consultant/specialist	34.6 (27)
**Teaching and Learning**		
Non-curricular physical activity programs	Both intramurals and non-competitive clubs	44.9 (35)
	Intramural only	19.2 (15)
	Non-competitive only	19.2 (15)
	None	16.7 (13)
**Healthy School Policy**		
Written policy	Yes	57.7 (45)
	No	30.8 (24)
	Missing	11.5 (9)
Use data from student health assessment to plan	Yes	38.5 (30)
	No	61.5 (48)
*Access during non-instructional time:*		
Indoor facilities	Yes	68.0 (53)
	No	32.1 (25)
Outdoor facilities	Yes	85.9 (67)
	No	11.5 (9)
	Missing	2.6 (2)
Equipment	Always	29.5 (23)
	Sometimes	59.0 (46)
	Never	11.5 (9)
*Access outside of school hours:*		
Gymnasium	Yes	78.2 (61)
	No	21.8 (17)
Indoor facilities	Yes	76.9 (60)
	No	21.8 (17)
	Missing	1.3 (1)
Outdoor facilities	Yes	84.6 (66)
	No	14.1 (11)
	Missing	1.3 (1)
Equipment	Yes	69.2 (54)
	No	29.5 (23)
	Missing	1.3 (1)
*School board provided resource:*		
Staff time	Yes	59.0 (46)
	No	34.6 (27)
	Missing	6.4 (5)
Additional space	Yes	32.1 (25)
	No	60.3 (47)
	Missing	7.7 (6)
Budget to improve health	≥$1001	34.6 (27)
	$1–$1000	15.4 (12)
	No funding	39.7 (31)
	Missing	10.3 (8)

Notes: PA = physical activity; SES = socioeconomic status. Due to rounding, the sum of the frequencies for a variable may not equal 100%.

**Table 2:** Weekly minutes of moderate-to-vigorous physical activity, by gender, for students attending schools that participated in Year 2 (2013–2014), Year 3 (2014–2015), and Year 4 (2015–2016) of the COMPASS Study in Ontario and Alberta, Canada.

Student Characteristics	Year 2 2013–2014 % (n)/Mean (SD)	Year 3 2014–2015 % (n)/Mean (SD)	Year 4 2015–2016 % (n)/Mean (SD)	3-Level Linear Mixed Effects Model (Unconditional Growth Model) β [95% CI]
**Female Students**	*n* = 7633	*n* = 7859	*n* = 6722	*n* = 22,223
**Cohort**				
Grade 9 in Y_2_	50.2 (3831)	52.0 (4088)	53.7 (3607)	-----
Grade 10 in Y_2_	49.8 (3802)	48.0 (3771)	46.3 (3115)	
**Ethnicity**				
White only	76.7 (5855)	76.5 (6011)	76.9 (5172)	-----
Other	23.3 (1778)	23.5 (1848)	23.1 (1550)	
**Moderate-to-vigorous physical activity**				
Minutes/week	785.5 (533.8)	734.8 (528.0)	704.6 (524.0)	Time: −39.89 [−46.48, −33.31] ***
**Male Students**	*n* = 7197	*n* = 7309	*n* = 6101	*n* = 20,597
**Cohort**				
Grade 9 in Y_2_	53.3 (3837)	54.9 (4012)	56.5 (3444)	-----
Grade 10 in Y_2_	46.7 (3360)	45.1 (3297)	43.6 (2657)	
**Ethnicity**				
White only	75.8 (5457)	75.0 (5484)	74.5 (4543)	-----
Other	24.2 (1740)	25.0 (1825)	25.5 (1558)	
**Moderate-to-vigorous physical activity**				
Minutes/week	961.8 (618.5)	946.0 (653.5)	912.4 (647.9)	Time: −18.03 [−26.25, −9.81] ***

Note: CI = confidence interval; *** *p* < 0.001.

**Table 3:** Fixed effects from longitudinal multilevel regression of student- and school-level variables associated with weekly minutes of MVPA in students attending schools that participated in Year 2 (2013–2014), Year 3 (2014–2015), and Year 4 (2015–2016) of the COMPASS Study in Ontario and Alberta, Canada.

		Female Students *n* = 9094 (*n* = 22,212 Repeated Measures)	Male Students *n* = 8567 (*n* = 20,607 Repeated Measures)
NULL MODEL		ICC	ICC
Repeated measures within students		0.450	0.460
Students nested within schools		0.019	0.024
**FULL MODEL**		**β [95% CI]**	**β [95% CI]**
**Level 1: Time**			
Year (continuous)		60.36 [−46.35, 167.07]	21.58 [−104.11, 147.27]
**Level 2: Student**			
Grade in Y_2_	Grade 9	REF	REF
	Grade 10	−66.90 [−84.83, −48.96] ***	−49.40 [−72.04, −26.75] ***
Ethnicity	White only	REF	REF
	Other	−51.19 [−84.83, −48.96] ***	−28.95 [−56.31, −1.59] *
**Level 3: School**			
**Social Environment**			
Enrolment	1–500	REF	REF
	501–1000	−5.62 [−54.61, 43.37]	45.65 [−18.72, 110.02]
	≥1001	−15.63 [−122.14, 90.88]	101.86 [−35.01, 238.73]
SES	25,000–50,000	REF	REF
	50,001–75,000	62.53 [−6.20, 131.26]	75.39 [−20.82, 165.61]
	75,001–100,000	5.06 [−76.14, 86.26]	37.27 [−66.89, 141.43]
	≥100,001	35.88 [−85.81, 157.56]	135.95 [−21.12, 293.01]
School Priority of PA	1st–3rd	REF	REF
	4th–6th	−0.57 [−54.17, 53.04]	1.20 [−67.09, 69.48]
	7th–10th	−1.56 [−79.46, 76.35]	−55.97 [−155.69, 43.75]
	Missing	−2.18 [−96.56, 92.20]	−36.76 [−159.01, 85.48]
Promotes PA events	Yes	−1.47 [−66.50, 63.58]	−25.66 [−105.46, 54.14]
**Physical Environment**			
Location	Rural/Small urban	REF	REF
	Medium urban	42.53 [−18.62, 103.69]	−16.14 [−97.26, 64.98]
	Large urban	1.70 [−67.30, 70.70]	−111.30 [−196.91, −25.69] *
Curtains available	Yes	1.10 [−40.19, 42.39]	7.87 [−48.69, 64.43]
Secure lockers available	Yes	−5.27 [−75.66, 65.12]	−12.86 [−101.18, 75.46]
Showers available	Yes	−75.97 [−143.93, −8.01] *	−52.06 [−141.89, 37.55]
**Partnerships and Services**			
Public health	Yes	41.10 [−2.49, 84.70]	−25.97 [−83.60, 31.65]
Non-governmental organization	Yes	5.46 [−34.58, 45.51]	−1.84 [−51.25, 47.58]
Parks and recreation	Yes	3.43 [−44.88, 51.73]	20.23 [−43.58, 84.04]
Youth organizations	Yes	43.89 [−6.56, 94.34]	23.32 [−42.18, 88.83]
Health and fitness club	Yes	−52.24 [−102.41, −2.08] *	−88.80 [−150.80, −26.79] **
Consultant/specialist	Yes	−38.07 [−86.17, 10.03]	57.66 [−4.28, 119.61]
**Teaching and Learning**			
Non-curricular PA programs	Intramural and non-competitive	−3.37 [−60.25, 53.51]	−8.99 [−85.68, 67.70]
	Intramural only	−12.32 [−78.23, 53.59]	16.34 [−68.18, 100.86]
	Non-competitive only	19.64 [−51.59, 90.87]	−27.91 [−119.29, 63.47]
	None	REF	REF
**Policy**			
Has written policy	Yes	−2.22 [−59.65, 55.21]	−32.29 [−101.49, 36.91]
Uses data to plan	Yes	6.20 [−39.43, 51.82]	−38.44 [−96.14, 19.26]
*Access during non-instructional time:*			
Indoor facility	Yes	−27.46 [−80.96, 26.03]	−10.25 [−78.41, 57.91]
Outdoor facility	Yes	77.32 [10.85, 143.79] *	8.35 [−79.31, 96.01]
Equipment	Always	103.24 [41.61, 164.86] ***	150.31 [70.96, 229.67] ***
	Sometimes	80.00 [7.65, 152.35] *	114.32 [24.85, 203.78] *
	Never	REF	REF
*Access after school hours:*			
Gymnasium	Yes	46.80 [−41.53, 135.14]	−13.97 [−116.64, 88.70]
Indoor facility	Yes	12.67 [−47.54, 72.89]	−18.01 [−95.95, 59.93]
Outdoor facility	Yes	−31.27 [−94.07, 31.53]	−21.53 [−103.71, 60.64]
Equipment	Yes	−56.70 [−129.31, 15.91]	−2.26 [−87.11, 82.59]
*Resources from school board:*			
Staff time	Yes	−4.89 [−50.60, 40.83]	−2.37 [−61.30, 56.56]
Space	Yes	−26.82 [−93.38, 39.74]	−20.03 [−102.04, 61.98]
Budget	No funding	REF	REF
	$1–$1000	30.49 [−27.81, 88.79]	92.93 [17.38, 168.48] *
	≥$1001	2.31 [−44.06, 48.69]	−37.19 [−93.48, 19.10]
	Other	12.47 [−62.48, 87.43]	−43.20 [−137.39, 51.00]
**Cross-level interactions (School-level variables and time)**			
**Social Environment**			
Enrolment * Time	1–500	REF	REF
	501–1000	30.04 [5.76, 54.32] *	−24.78 [−55.39, 5.82]
	≥1001	19.85 [−32.99, 72.69]	−74.12 [−139.25, −8.98] *
SES * Time	25,000–50,000	REF	REF
	50,001–75,000	−43.80 [−76.50, −11.09] **	−49.45 [−91.85, −7.05] *
	75,001–100,000	−67.87 [−107.58, −28.15] ***	−68.40 [−117.01, −19.80] **
	≥100,001	−6.25 [−64.14, 51.64]	−74.29 [−147.34, −1.24] *
School Priority of PA * Time	1st–3rd	REF	REF
	4th–6th	−38.71 [−65.14, −12.28] **	19.27 [−13.49, 52.03]
	7th–10th	−5.82 [−44.28, 32.64]	83.13 [36.18, 130.08] ***
	Missing	−23.42 [−69.99, 23.14]	72.16 [14.60, 129.72] *
Promotes PA events * Time	Yes	−33.28 [−65.10, −1.45] *	−2.88 [−39.88, 34.11]
**Physical Environment**			
Location * Time	Rural/Small urban	REF	REF
	Medium urban	−25.25 [−54.87, 4.37]	−12.35 [−50.89, 26.20]
	Large urban	−27.93 [−61.75, 5.88]	8.99 [−31.81, 49.78]
Curtains available * Time	Yes	−1.45 [−21.38, 18.48]	2.98 [−22.65, 28.62]
Secure lockers available * Time	Yes	1.70 [−34.30, 37.70]	−30.51 [−73.94, 12.91]
Showers available * Time	Yes	21.71 [−12.24, 55.66]	21.33 [−22.12, 64.78]
**Partnerships and Services**			
Public health * Time	Yes	−44.96 [−65.53, −24.38] ***	3.55 [−22.99, 30.09]
Non-governmental organization * Time	Yes	−17.57 [−37.15, 2.01]	−0.95 [−24.25, 22.35]
Parks and recreation * Time	Yes	−1.94 [−25.33, 21.45]	6.48 [−23.48, 36.45]
Youth organizations * Time	Yes	−17.85 [−42.56, 6.87]	−21.68 [−53.30, 9.93]
Health and fitness club * Time	Yes	24.75 [−0.41, 49.92]	41.54 [11.91, 71.18] **
Consultant/specialist * Time	Yes	32.44 [8.93, 55.94] **	−6.95 [−35.80, 21.90]
**Teaching and Learning**			
Non-curricular PA programs * Time	Intramural and non-competitive	13.49 [−14.50, 41.49]	−18.06 [−54.75, 18.62]
	Intramural only	4.50 [−28.59, 37.59]	−24.56 [−65.48, 16.36]
	Non-competitive only	2.64 [−32.66, 37.95]	5.16 [−38.76, 49.09]
	None	REF	REF
**Policy**			
Has written policy * Time	Yes	−0.05 [−28.52, 28.43]	−7.21 [−39.86, 25.44]
Uses data to plan * Time	Yes	5.29 [−17.58, 28.43]	38.98 [11.31, 66.64] **
*Access during non-instructional time:*			
Indoor facility * Time	Yes	33.35 [6.46, 60.23] *	17.27 [−15.88, 50.42]
Outdoor facility * Time	Yes	−74.83 [−107.32, −42.34] ***	−23.03 [−64.51, 18.45]
Equipment * Time	Always	3.43 [−25.13, 31.99]	−36.13 [−70.98, −1.29] *
	Sometimes	11.86 [−22.60, 46.33]	−48.61 [−88.31, −8.92] *
	Never	REF	REF
*Access after school hours:*			
Gymnasium * Time	Yes	−20.71 [−63.64, 22.22]	15.35 [−32.00, 62.69]
Indoor facility * Time	Yes	−11.35 [−39.84, 17.15]	−21.99 [−57.65, 13.67]
Outdoor facility * Time	Yes	3.23 [−26.47, 32.92]	28.68 [−8.70, 66.06]
Equipment * Time	Yes	1.64 [−33.72, 37.01]	3.35 [−35.19, 41.88]
*Resources from school board:*			
Staff time * Time	Yes	36.49 [13.87, 59.11] **	−3.02 [−31.07, 25.03]
Space * Time	Yes	−35.58 [−68.90, −2.27] *	41.32 [2.26, 80.37] *
Budget * Time	No funding	REF	REF
	$1–$1000	19.10 [−9.01, 47.20]	−36.42 [−72.17, −0.68] *
	≥$1001	41.11 [18.82, 63.40] ***	11.02 [−14.93, 36.97]
	Other	21.51 [−14.54, 57.55]	62.07 [17.92, 106.22] **

Notes: Unless otherwise stated, the reference category is any response other than a definitive “Yes” (i.e., no, not applicable, no response, uncodeable); ICC = intraclass correlation; CI = confidence interval; PA = physical activity; SES = socioeconomic status; * *p* < 0.05; ** *p* < 0.01; *** *p* < 0.001.

## Data Availability

The datasets generated and analyzed for this study will not currently be shared because this is an ongoing study; however, access to the data supporting the findings of this study can be requested at https://uwaterloo.ca/compass-system/information-researchers (accessed on 20 October 2021).
